# Sodium butyrate improves antioxidant stability in sub-acute ruminal acidosis in dairy goats

**DOI:** 10.1186/s12917-018-1591-0

**Published:** 2018-09-10

**Authors:** Nana Ma, Juma Ahamed Abaker, Muhammad Shahid Bilal, Hongyu Dai, Xiangzhen Shen

**Affiliations:** College of Veterinary Medicine, Nanjing Agricultural University, Nanjing, 210095 People’s Republic of China

**Keywords:** Oxidative stress, High-grain diet, Sodium butyrate, Dairy goats

## Abstract

**Background:**

Currently, little is known about the effect of sodium butyrate (NaB) on oxidative stress following grain-induced sub-acute ruminal acidosis in dairy goats. In the present study, 18 lactating dairy goats implanted with a ruminal cannula and permanent indwelling catheters in the portal and hepatic veins were randomly allocated into 3 treatment groups over 20 weeks: low grain (**LG**, 40% grain; *n* = 6), high grain (**HG**, 60% grain; *n* = 6) and high grain with sodium butyrate (**HG + NaB**, 60% grain + NaB; *n* = 6).

**Results:**

When added to the HG diet, NaB increased the mean ruminal pH and reduced the levels of ruminal, portal and hepatic LPS; Additionally, we observed an increase in SOD1, SOD2, SOD3, GPX1 and CAT mRNA expression, increased levels of TSOD and CAT enzyme activity as well as increased total antioxidant capacity (T-AOC) and decreased malondialdehyde (MDA) in both the liver and plasma, while GPx activity increased in the liver of goats fed the HG + NaB diet. The mRNA expression of UGT1A1, NQO1, MGST3, and Nrf2, as well as total Nrf2 protein levels were increased in goats fed the HG + NaB diet.

**Conclusions:**

Our study indicates that sodium butyrate could improve the oxidative status in sub-acute ruminal acidosis through the partial activation of Nrf2-dependent genes.

## Background

Oxidative stress is an active research area in veterinary medicine, and it is implicated in the pathogenesis of many pathological conditions, including sepsis, mastitis, enteritis, pneumonia, respiratory and joint diseases [1] and hepatic insufficiency [2]. Moreover, oxidative stress is believed to play a significant role in regulating the metabolic activity of some organs and in productivity in farm animals [3]. In response to oxidative stress, organisms can redirect their metabolic flux from glycolysis to pentose phosphate pathway which provides the reducing power for the major cellular redox systems [4]. Pregnancy and lactation are physiological stages considered to induce some metabolic stress [5]. Recent studies have focused on metabolic diseases that affect dairy ruminants during the peripartum period [6]. Subacute ruminal acidosis (SARA) is a common metabolic disorder in high-producing dairy farms that leads to great economic losses [7]. In ruminants, SARA is a metabolic disease induced by feeding excessive amounts of highly fermentable carbohydrate and insufficient dietary coarse fibre [8]. Lactating goats were reported to have experienced a certain stress during subacute ruminal acidosis [9]. As a response to the demand for increased milk production and food scarcity, grain is commonly used to combat negative energy balance [10]. However, increasing grain-to-forage ratios leads to depression of ruminal pH [8, 11]. The accumulation of short chain fatty acids (SCFA) leads to SARA and affects the integrity of the rumen epithelium [11]. When SARA compromises epithelial integrity, it results in health problems such as liver abscesses and even laminitis [12].

Lipopolysaccharide (LPS) is a bacterial product reported to be translocated to the blood circulation when the gastrointestinal wall and epithelial tight junctions are perturbed [13]. Translocation of LPS into blood circulation in SARA was found to trigger an inflammatory response and leads to oxidative stress [13–15], due to an increase of reactive oxygen species (ROS), associated with unbalanced dietary anti-oxidants [16]. The alteration of oxidative balance could induce oxidative stress and, consequently, cellular damage if not balanced with antioxidants [17].

Butyrate, a short-chain fatty acid, is a major microbial fermentation product of dietary fibre in the forestomach (rumen) of ruminants that contributes to approximately 70% of the daily metabolizable energy requirement of ruminants [18, 19]. Butyrate is known to maintain a barrier function that regulates several cellular processes and to modulate gene expression through histone deacetylase complex (HDAC) inhibition [20]. Butyrate shows a protective effect against oxidative DNA damage induced by H2O2 in isolated human colonocytes and in HT29 tumour cells [21]. Previous studies in ruminants showed that ingested butyrate could down-regulate the expression of virulence genes, increase ration digestibility, increase growth rate, and decrease oxidative stress and cytokine synthesis, even at very low doses [22, 23], resulting in control of the pathogen. Feeding sodium butyrate to lipopolysaccharide-challenged chickens increases serum superoxide dismutase (SOD) activity and decreases malondialdehyde (MDA) content in raised chickens [24]. LPS is believed to be a key point in the pathogenesis of SARA [25], whose diagnosis is laborious, but proper management and feeding can prevent SARA [25]. However, no information is currently available about the effects of sodium butyrate on stress parameters of ruminants fed a high-grain diet (HG). Therefore, the objective of this study is to examine the effect of adding sodium butyrate to a high-grain diet formula (HG + NaB) on antioxidant function and to determine whether NaB could prevent translocation of LPS into the blood and alleviate oxidative stress during SARA. We hypothesized that compared with the low-grain diet (LG), the high-grain diet (HG) would increase endotoxin concentrations and induce oxidative stress responses in lactating dairy goats as a result of decreased ruminal pH and that sodium butyrate (NaB) could stimulate fibre-digesting bacteria and consequently stabilize rumen pH and rumen LPS and prevent oxidative stress.

## Methods

### Goats and diets

Eighteen lactating Saanen dairy goats (average bodyweight, 38.9 ± 2.06 kg) in parity 1 or 2 (milk yield, 1.13 ± 0.0200 kg/day) were selected based on lactation time and were randomly assigned to three groups. All goats were ruminally cannulated, hepatic catheters were fitted surgically in the hepatic vein and portal vein, then exteriorized and fixed on the para-lumbar area beneath the transverse process. The first group (*n.* = 6; LG) was given a low-grain diet with a 4:6 grain-to-forage ratio, the second group (*n.* = 6; HG) received a high-grain diet with a 6:4 grain-to-forage ratio, and the third group (*n.* = 6; HG+ NaB) was given the high-grain diet mixed with sodium butyrate. Goats were housed in separate boxes and given food twice/day, at 8:00 am and at 18:00 pm. Dry matter intake was 1.9 ± 0.15 kg/d/head, with free access to water throughout the experiment. The goats were observed daily regarding feed intake, rectal temperature and respiratory rate. The ingredients and nutritional composition of the diets are presented in Table 1.

**Table 1:** The Ingredients of the diets and nutritional composition

Ingredients, % of DM	High grain (HG)^a^	High grain with NaB (HG + NaB) ^a^	Low grain (LG) ^a^
Corn (g/100 g)	23.07	23.07	29.2
Bran (g/100 g)	28.03	28.03	0
Soybean meal (g/100 g)	2	2	8.43
Rape seed meal (g/100 g)	3.7	3.7	0
Stone powder (g/100 g)	1.43	1.43	0.57
Calcium hydrophosphate (g/100 g)	0.6	0.6	0.9
Premix^b^ (g/100 g)	0.4	0.4	0.5
Salt (g/100 g)	0.5	0.5	0.4
Sodium butyrate (g/100 g)	–	1	–
Coarse material			0.00
Oats grass (g/100 g)	32	32	48
Alfalfa Oats grass (g/100 g)	8	8	12
Nutritional index			
NE (MJ/kg)	17.56	17.56	17.17
Fat (%)	3.6	3.6	2.39
CP (%)	16.76	16.76	17.79
Starch	19.914	19.914	17.295
NDF (%)	42.64	42.64	40.79
ADF (%)	7.22	7.22	2.83
Ca (%)	1.55	1.55	1.73
P (%)	0.76	0.76	0.79

*NE* Net Energy, *CP* Crude Protein, *NDF* Neutral Detergent Fibre, *ADF* Acid Detergent Fibre, *Ca* Calcium, *P* Phosphorus

^a^Treatment LG, 40% grain on DM basis; HG, 60% grain on DM basis, HG, 60% grain on DM basis + sodium butyrate

^b^Premix consists of the following ingredients per kg of diet: vitamin A, 6000 IU; vitamin D, 2500 IU; vitamin E, 53.6 IU; Cu, 6.25 IU/kg; Fe, 62.5 mg; Zn, 62.5 mg; Mn, 50 mg; mg I, 0.125 mg; Co, 0.125 mg; Mo, 0.125 mg

Blood samples and rumen fluid were collected from the three groups on the last three consecutive days of the 20th week. Blood was sampled from the jugular vein in 5 mL vacuum tubes containing sodium heparin as an anticoagulant. Plasma was separated by centrifugation at 3000×g at 4 °C for 15 min, and aliquots were frozen at − 20 °C for the analysis of LPS, thiobarbituric acid reactive substances (MDA), total antioxidant capacity (T-AOC) and antioxidant enzyme activity.

Rumen fluid was collected through a rumen fistula every 2 h for 7 h after feeding. The samples were centrifuged at 10,000×g for 45 min, and the supernatant was aspirated and filtered through a disposable 0.22-μm filter. The filtrate was collected in a sterile depyrogenated glass tube (previously heated at 250 °C for 2 h) and heated at 100 °C for 30 min. Samples were cooled at room temperature for 15 min and stored at − 20 °C for LPS measurements. The pH of the ruminal fluid was immediately detected using a pH meter (Sartorius, Basic pH Meter PB-10, PB-21, Goettingen, Germany). The goats were slaughtered after the 20th week after fasting overnight and liver samples were collected, immediately frozen in liquid nitrogen and stored at − 70 °C.

### Volatile fatty acids analysis

Chromatography with FFAP 123–3233 30 m × 0.32 mm × 0.5 μm capillary column (Agilent Technologies, Stevens Creek Blvd, Santa Clara, CA, United States) in an Agilent 7890A system (Agilent Technologies) was used to assay the VFA concentration as previously described by Wood et al., (2010) [26].

### Milk analysis

Goats were milked twice a day at 8:00 am and at 18:00 pm and milk yield were recorded daily. The milk sample was taken to assay the milk fat and milk protein concentrations using MilkoScan (FT1, Foss, Hillerod, Denmark).

### Measurement of LPS

The concentration of LPS in the rumen fluid, portal vein, hepatic vein plasma was determined using a chromogenic endpoint limulus amoebocyte lysate assay kit (CE64406 & CE80545, Chinese Horseshoe Crab Reagent Manufactory Co., Ltd., Xiamen, China) as described by Dong et al. [27] to detect LPS concentrations in the ruminal fluid and plasma with a minimum detection limit of 0.01 EU/mL (EU, Endotoxin Unit).

### Radioimmunoassay

Radioimmunoassay was applied to determine the concentrations of master cytokines, including IL-1β, IL-6 and TNF-α in circulating blood. The concentrations of IL-1β, IL-6 and TNF-α were determined with commercially available human radioimmunoassay kits purchased from Beijing North Institute of Biological Technology. The detected range of radioimmunoassay kits for IL-1β (cat. C09DJB), IL-6 (cat. C12DJB) and TNF-α (cat. C06PJB) were 0.1–8.1 ng/mL, 50–4000 pg/ml and 9–590 fmol/mL, respectively.

### Quantitative real time-PCR (qRT-PCR)

Total RNA was prepared from 50 mg liver tissue using TRIZOL (Invitrogen, USA) as described by the manufacturer’s protocol. RNA quality was assessed by both agarose gel (1%) electrophoresis and a NanoDrop ND-1000 Spectrophotometer (Thermo Scientific, USA). Only samples with an A260 to A280 ratio between 1.8 and 2.1 were used in subsequent experiments. Reverse transcription (RT) was performed using 250 ng/μL RNA to synthesize cDNA with PrimeScript RT Master Mix Perfect Real Time (Takara Co., Otsu, Japan) according to the manufacturer’s instructions.

Primers for the CAT, GPX1, GPx3, SOD1, SOD2, and SOD3 genes and for GAPDH as a reference gene were designed using the NCBI primer blast online software. Each cDNA sample was amplified using SYBR Green (Takara Co., Otsu, Japan) and an ABI 7300 Fast Real-time PCR System (Applied Bio system, USA). Briefly, the reaction mixture consisted of 2 μL of cDNA and 0.4 μM primers, 10 μL of SYBR green, 0.4 μL of ROX, and 6.8 mL of ddH_2_O in a final volume of 20 μL. The qRT-PCR conditions were as follows: denaturation at 95 °C for 15 s, followed by 40 cycles of annealing at 60 °C for 31 s and extension at 72 °C. All the reactions were run in triplicate, and the specific PCR products were confirmed by gel electrophoresis on a 3.0% agarose gel. The primer sequences and the accession numbers of targeted genes are provided in Table 2.

**Table 2:** Primer sequences used for real-time PCR amplification of selected genes in the livers of lactating goats

Gene^a^	Primers 5′ to 3’^b^	Size(bp)	Accession ^c^
SOD1	ACACAAGGCTGTACCAGTGC TTCACATTGCCCAGGTCTCC	104	NM_001285550.1
SOD2	TCAATAAGGAGCAGGGACGC AGCAGGGGGATAAGACCTGT	85	XM_005684984.1
SOD3	ATCGACCCGAACGGTAACGC AGGACATAGAAGGGGTCTGCG	199	XM_005681678.1
CAT	ACAATGTCACTCAGGTGCGG TCTCACACAGGCGTTTCCTC	70	XM_005690077.1
GPx1	GTAGCATCGCTCTGAGGCAC TTGGCATTTTCCTGATGCCC	131	XM_005695962.1
GPx3	TTCGGTCTGGTCATTCTGGG CGAACATACTTGAGGGTGGC	95	XM_005683183.1
HMOX2	AGCTAGACAAAGGTGCCCTG CAGGATGCTGACAGGCAAAG	113	XM_005697930.1
MGST3	AGCTCACCAGAACACGTTGG GCCCAAACCAGAAGCTATGC	94	NM_001285615.1
NQO1	CATGGCTGTCAGAAAAGCACTG GACACAGTGACCTCCCATCC	120	XM_005692193.1
UGT1A1	TCCGGAGCAGAAAGCTATGG CCGAGTCTTTGGGTGACCAAG	160	XM_005678746.1
TXNRD1	TTTCGCTCAGTTTGCTCCAG TCCTGAGAAGCCTTCAGGGTC	200	XM_005680564.1
NF-kB	AGGTGGCGATCGTTGTTCTA TTGCCTTTGTTCTTCCTGCC	226	XM_00569893.1
NrF2	AGCCAGGTGAGATGGAACTG CCAGACTCCCTGTTTCGCTG	120	XM_005679848.2
MT2A	TCCTGCAAGAAGAGCTGCTG AACTGCACTTGTCCGAGGC	94	XM_005691999.1
SRXN1	GACACGATCCGGGAGAATCC GGTCTGAGAGGGTGGATTGG	175	XM_013968785.1
MT1A	ATAATAGCGCTCGGCTCCTG TTGGAGGAAAAGCGAGGTCC	98	XM_005692001.2
GAPDH	CCTGCCCGTTCGACAGATAG CCGTTCTCTGCCTTGACTGT	249	XM_005680968.2

^a^*SOD1* superoxide dismutase 1, *SOD2* superoxide dismutase 2, *SOD3* superoxide dismutase3, *GPX1* glutathione peroxidase1, *GPX3* glutathione peroxidase 3, *CAT* catalase, *HMOX2* haem oxygenase 2, *MGST3* microsomal glutathione S-transferase 3, *MT1A* metallothionein 1A, *NQO1* NAD(P)H, quinone 1, *UGT1A1* UDP glucuronosyltransferase 1 family, polypeptide A1, *MT2A* metallothionein 2A, *TXNRD1* thioredoxin reductase 1, *SRXN1* sulfiredoxin 1, *GAPDH* glyceraldehyde 3-phosphate dehydrogenase

^b^The first primer listed for each gene is the forward primer, and the second primer is the reverse primer

^c^The reference sequence numbers are given for primers whose source is the National Center for Biotechnology Information (NCBI) GenBank database (http://www.ncbi.nlm.nih.gov/genbank/)

The data were normalized to the mean of the housekeeping gene *GAPDH* to control for variability in expression levels and were analysed using the 2^-ΔΔCt^ method ((Ct _target gene_ - Ct _*GAPDH*_) treatment - (Ct _target gene_ - Ct _*GAPDH*_) control) as previously described [28].

### Oxidative and antioxidative biomarker analysis in liver and plasma

The antioxidant assays were conducted using assay kits purchased from the Nanjing Jiancheng Institute of Bioengineering (Nanjing, Jiangsu, China), and the assays were conducted according to the method described by Zheng et al. [29]. Briefly, one gram of frozen liver tissue in 10 ml of homogenization buffer (0.9% cool physiological saline) was homogenized on ice for 30 s at 12500 rpm using a Polytron PT 1200 E (POLYTRON® PT 1200 E Manual Disperser, Luzern - Switzerland). The homogenate was centrifuged at 2500 rpm for 10 min at 4 °C, and the supernatant (10% concentration) was aliquoted. Physiological saline (0.9%) was used to dilute the supernatant to different concentrations, which was stored at − 20 °C for the following analyses:

### Glutathione peroxidase

A Nanjing Jiancheng Bioengineering Institute commercial kit (Nanjing, Jiangsu, China) was used to determine GPx spectrophotometrically (UV3600, Daojin Corp., Japan) at 412 nm as previously described by Wu et al. [30] the oxidizing speed of GSH was expressed as the GSH reduction within a certain time. One unit of GPx activity was defined as 1 μmol/L GSH oxidized to glutathione disulfide (GSSG)/mg protein/ min.

### Superoxide dismutase

Plasma and liver homogenate were used to determine SOD activity based on a reaction system that contained xanthine and xanthine oxidase using Nanjing Jiancheng Bioengineering Institute commercial kits (Nanjing, Jiangsu, China, Cat. No. A001–1 SOD). The superoxide anion (O-·2) oxidizes hydroxylamine to form nitrite and was assayed spectrophotometrically (UV3600, Daojin Corp., Japan) at 550 nm. One unit of SOD is defined as the amount of sample resulting in 50% inhibition of nitroblue tetrazolium reduction.

### Catalase

Nanjing Jiancheng Bioengineering Institute commercial kits (Nanjing, Jiangsu, China) were used to determine catalase activity based on the decrease in H_2_O_2_ concentration in 15 s as previously described by Wang et al. [31]. Catalase was assayed spectrophotometrically (UV3600, Daojin Corp., Japan) at 405 nm. One unit of CAT activity was defined as the amount of enzyme decomposing 1 mol of H_2_O_2_ in 1 s.

### Malondialdehyde (MDA) measurement

MDA levels were measured spectrophotometrically at 532 nm using the thiobarbituric acid reaction method as previously described by Chen et al. [32] using commercially available kits (Nanjing Jiancheng Bioengineering Institute). Lipid peroxidation was expressed as nmol MDA/mg protein or nmol MDA/mL plasma.

### Determination of total antioxidants (T-AOC)

T-AOC was measured spectrophotometrically based on the reduction of Fe3+ to Fe2+, which combines with phenanthrene and forms a coloured compound that can be detected at 520 nm. One unit of T-AOC was defined as the extent to which the optical density is increased by 0.01 /mg protein or/mL plasma/min.

### Western blotting analysis

Proteins were extracted from 100 mg liver tissue with RIPA buffer (Beyotime Biotechnology Co., Ltd., Shanghai, China). Protein concentrations were assayed using a bicinchoninic acid (BCA) protein assay kit (Pierce, Rockford, IL, USA). The concentration was normalized to 4 μg/μL, and 10 μL protein in sodium dodecyl sulfate (SDS) loading buffer was subjected to 10% SDS-polyacrylamide gel electrophoresis (PAGE) and transferred to nitrocellulose (NC) membranes (Millipore, Danvers, MA) at 4 °C for 90 min; 10% skim milk in Tris-buffered saline was used to block the membrane. Monoclonal goat antibodies against Nrf2 and GAPDH (Santa Cruz Biotechnology, CA, USA, 1:200 dilution) (Bioworld, CA, USA, 1:10,000 dilution) were used at 4 °C overnight, and then the membrane was washed and treated with horseradish peroxidase-conjugated donkey anti-goat secondary antibody. Immunoreactive proteins were detected by chemiluminescence using an ECL reagent (Super Signal West Pico Trial Kit, Pierce, USA) and subsequently analysed by autoradiography. Quantitation of the results was performed using a Bio-Rad Gel Doc 2000™ System (Bio-Rad, USA) and analysis with Bio-Rad TDS Quantity One software (Bio-Rad). The relative quantities of proteins were determined by a densitometer and expressed as absorbance units (AU).

### Statistical analysis

All the data were analysed using SAS software (SAS version 9.2, SAS Institute Inc., USA); the normality of the distribution of variables was tested by the Shapiro–Wilk test. The mixed procedure of SAS was used to analyse pH and milk data with a repeated measures design. The effects of diet and time were considered fixed factors. The effects of goats were considered random. The time within treatments and goats were considered repeated measurements, and compound symmetry (CS) was used as the type of covariance. Differences between groups regarding ruminal volatile fatty acids and proinflammatory cytokines, gene expression, enzyme activity and other biomarkers were analyzed by analysis of variance (ANOVA) followed by post hoc Dunnett’s multiple comparison tests. Differences were considered significant when *p* values ≤0.05. Graphs were made using Graph Pad Prism version 5.00 (Graph Pad Software).

## Results

### Ruminal pH

**Fig. 1:**
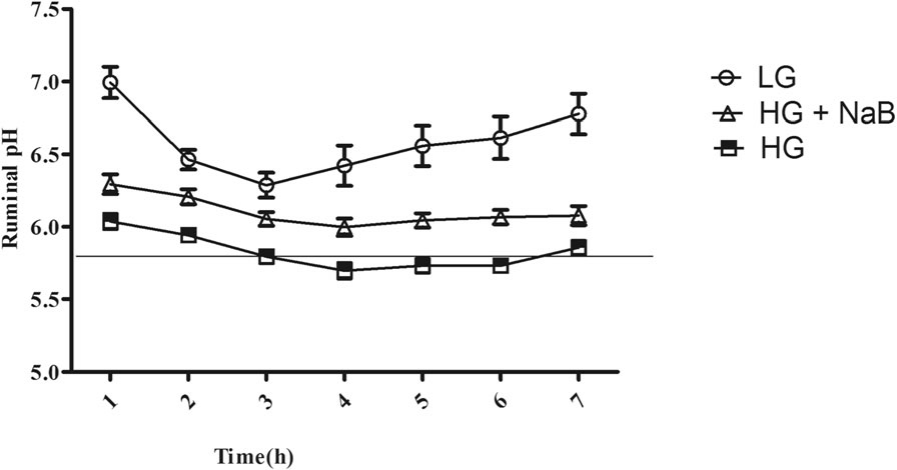
Comparison of pH values in ruminal fluid among the low-grain (LG), high-grain (HG) and high-grain with sodium butyrate (HG + NaB) groups. Ruminal fluid samples were collected at times ranging from 0 to 7 h for three consecutive days during the 20th week. Data were analysed by mixed models in SAS (SAS version 9.2, SAS Institute Inc., USA) and are expressed as the mean ± S.E.M. Significant differences were observed at all sampling times (*p* < 0.05). The error bars indicate the standard error of the mean

As shown in Fig. 1, significant differences were observed in ruminal pH among the HG diet, HG + NaB and LG diet groups (*p* < 0.05); NaB added to the HG diet significantly improved rumen pH compared to the HG diet alone; however, rumen pH still remained significantly lower than in the LG diet.

### Milk yield and composition

The results revealed that the milk yield, milk fat %, the milk fat yield, milk protein % and protein yield were significantly decreased in goats fed HG compared with HG + NaB and goats fed LG (*p* = 0.01, *p* = 0.012, *p* = 0.001, *p* = 0.024, and *p* = 0. 004 respectively, Table 3).

**Table 3:** The effect of Sodium butyrate on milk yields and milk composition of goats fed HG diet versus those fed HG alone and LG diet

	Treatment^d^				*p* value		
Item	LG	HG	HG + NaB	SEM	Diet	week	Diet*week
Milk yield	0.962^a^	0.941^b^	1.032^c^	0.07	0.01	0.01	0.01
Fats %	3.365^a^	2.852^b^	3.275^a^	0.128	0.012	0.35	0.3
Fats yield, kg	0.034^a^	0.025^b^	0.035^a^	0.001	0.001	0.02	0.56
Protein %	4.192 ^a^	3.801^b^	4.106 ^a^	0.064	0.024	0.01	0.134
Protein yield, kg	0.043^a^	0.035^b^	0.044^a^	0.001	0.004	0.002	0.09

The milk yield and milk compositions were analyzed by the mixed model of SAS with repeated measures, *p* ≤ 0.05 was considered significant

^a, b and c.^ The means with different superscript were considered significantly different

^d^*LG* low gain, *HG* high grain, *HG + NaB* high grain + sodium butyrate

### LPS content in ruminal fluid, hepatic vein plasma and portal vein plasma

**Fig. 2:**
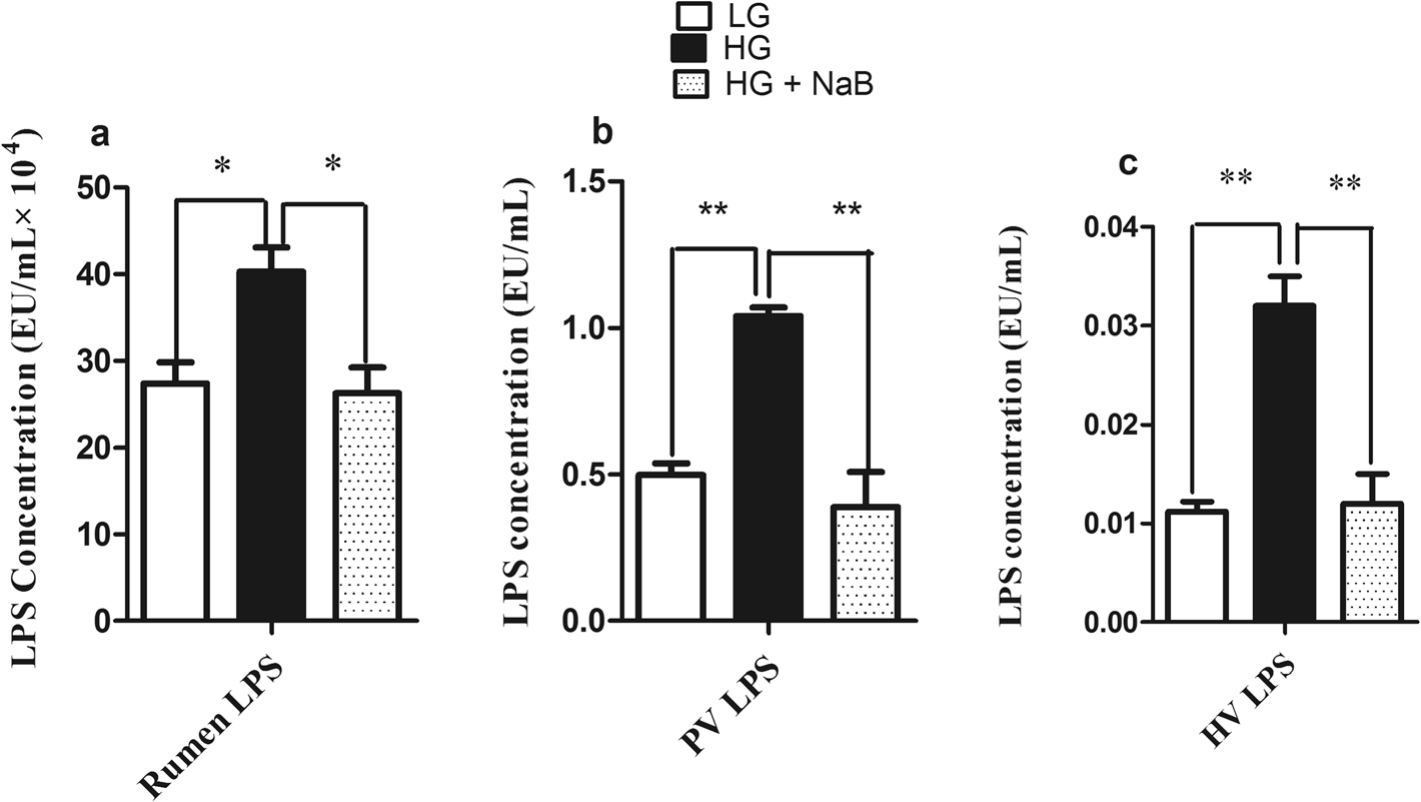
Effect of sodium butyrate on LPS concentration in the rumen, portal vein, hepatic vein and jugular vein plasma of dairy goats fed three diets (LG, HG and HG + NaB). **a** Ruminal LPS, **b** Portal vein LPS, **c** Hepatic vein LPS. The concentration of lipopolysaccharide (LPS) in plasma was determined by a chromogenic endpoint limulus amoebocyte lysate assay kit (CE64406 &CE80545, Chinese Horseshoe Crab Reagent Manufactory Co., Ltd., Xiamen, China). Data were analysed by mixed model in SAS (SAS version 9.2, SAS Institute Inc., USA) and are expressed as the mean ± S.E.M. **P < 0.01, * *P* < 0.05. LG = low grain; HG = high grain; HG + NaB = high grain + sodium butyrate

The concentration of LPS in each of the 3 groups is presented in Fig. 2. The highest LPS concentration in the rumen fluid was observed in goats fed the HG diet (*p* ≤ 0.01), compared to those fed the LG and HG diets with sodium butyrate. Consistent with the ruminal LPS concentration, goats fed the HG diet had a higher level of LPS in their portal vein plasma than those in the other groups (*p* ≤ 0.01), whereas no differences were observed between the LG diet and the HG diet with NaB. Moreover, the LPS concentration in hepatic vein plasma showed a significant difference among the three groups (*p* ≤ 0.01).

### Levels of pro -inflammatory cytokines in the peripheral blood

The results are shown in Table 4. The concentrations of IL-1β, IL-6 and TNF-α were remarkably increased (*p* = 0.024, *p* = 0.003 and *p* = 0.01 respectively) in goat fed high grain diet. However, sodium butyrate significantly decreases these cytokines when added to HG diet.

**Table 4:** The effect of Sodium butyrate on proinflammatory cytokines in goats fed HG diet versus those fed HG alone and LG diet

	Treatment				
Item	LG	HG	HG + NaB	SEM	*p* value
IL-1β, ng/mL	0.1568^a^	0.2500^b^	0.1670^a^	0.024	0.003
IL-6, pg/mL	289.01^a^	612.5^b^	404.8^c^	25.40	0.01
TNF-α fmol/mL	4.008^a^	7.236^b^	2.728^c^	0.234	0.007

The milk yield and milk compositions were analyzed by the mixed model of SAS

*LG* low gain, *HG* high grain, *HG + NaB* high grain + sodium butyrate

^a, b and c^The means with different superscript were considered significant different

### Rumen volatiles fatty acids

**Fig. 3:**
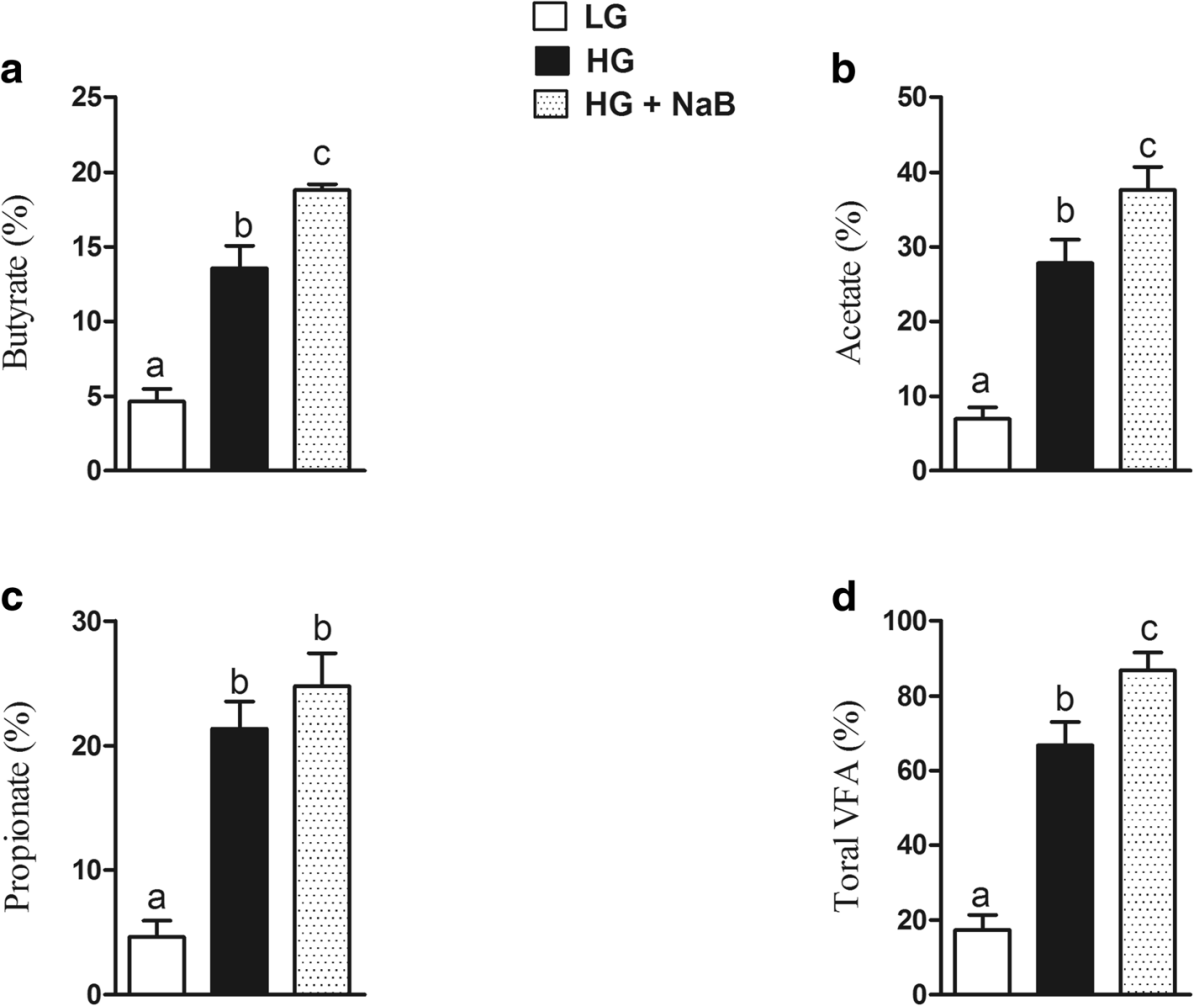
Effect of sodium butyrate on volatile fatty acids on ruminal fluid of goats fed HG with sodium butyrate compared with high grain alone and low grain. **a** Butyrate. **b** Acetate. **c** Propionate. **d** Total volatile fatty acids. Data were analyzed by the mixed model of SAS (SAS version 9.2, SAS Institute Inc), and expressed as the mean ± S.E.M. The means with different superscript were considered significantly different

The results of ruminal VFA concentrations were shown in Fig. 3. The levels of butyrate, propionate, and total VFA were significantly higher in the HG + NaB group than the HG group, meanwhile LG diet showed the lowest values of ruminal VFA (*p* = 0.01, *p* = 0.001, *p* = 0.001).

### Antioxidant gene expression in goat liver tissue

**Fig. 4:**
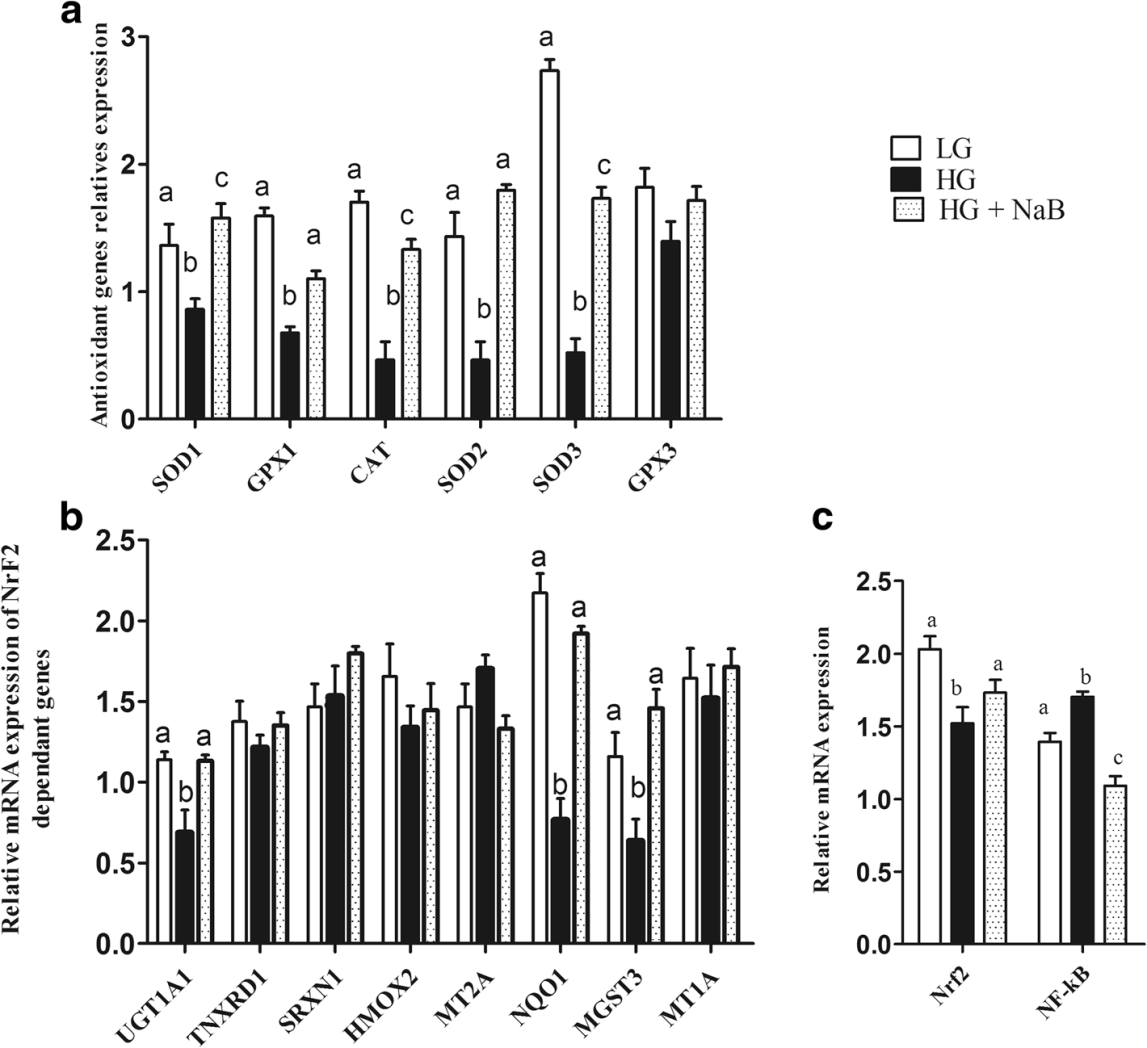
Effect of sodium butyrate on relative mRNA expression in the liver of goats fed HG vs. HG alone and LG. Gene expression was analysed quantitative real time-PCR (qRT-PCR). **a** mRNA abundance of genes encoding antioxidant enzymes in the liver (**b**) mRNA abundance of Nrf2 target genes in the liver (**c**) NrfF2 and NF-kB mRNA expression. *GAPDH* was used as the reference gene for gene expression. Data were analysed by mixed model in SAS (SAS version 9.2, SAS Institute Inc., USA) and are expressed as the mean ± S.E.M. **P < 0.01, * *P* < 0.05. LG = low grain; HG = high grain; HG + NaB = high grain + sodium butyrate; SOD1 = superoxide dismutase 1; SOD2 = superoxide dismutase 2; SOD3 = superoxide dismutase 3; GPX1 = glutathione peroxidase1; GPX3 = glutathione peroxidase 3; CAT = catalase; HMOX2 = haem oxygenase 2; MGST3 = microsomal glutathione S-transferase 3; MT1A = metallothionein 1A; NQO1 = NAD(P)H dehydrogenase, quinone 1; UGT1A1 = UDP glucuronosyltransferase 1 family, polypeptide A1; MT1E = metallothionein 1E; MT2A = metallothionein 2A; TXNRD1 = thioredoxin reductase 1; SRXN1 = sulfiredoxin 1; NrfF2 = nuclear factor E2-related factor 2; NF-kB = nuclear factor kappa B; *GAPDH =* glyceraldehyde 3-phosphate dehydrogenase

Among the antioxidant genes that were evaluated, including SOD1, SOD2, SOD3, GPX1, GPX3 and CAT, significant increases in the expression of SOD1, SOD2, SOD3, GPX1 and CAT in the HG + NaB group (*p* = 0.008; *p* = 0.001; *p* = 0.001; *p* = 0.001 and *p* = 0.001, respectively) were observed (Fig. 4a), whereas in the HG group, lower levels of these genes were expressed than in the LG diet group. No significant differences were noticed between goats fed the HG + NaB and LG diets except for CAT, GPX1 and SOD3.

The mRNA expression levels of hepatic UGT1A1, NQO1 and MGST3 were significantly increased with the HG + NaB diet (*p* = 0.008; *p* = 0.001 and *p* = 0.002, respectively). Conversely, these genes were significantly decreased in the HG diet group compared to the LG and HG + NaB diets. The expression levels of hepatic TNXRD1, HMOX2, MT1E, MT1A, MT2A, and SRXN1 showed no difference among the three groups (Fig. 4b). NF-κB and Nrf2 mRNA were significantly different among the three groups (*p* ≤ 0.01) (Fig. 4c).

### Total antioxidant capacity and lipid peroxidation

**Fig. 5:**
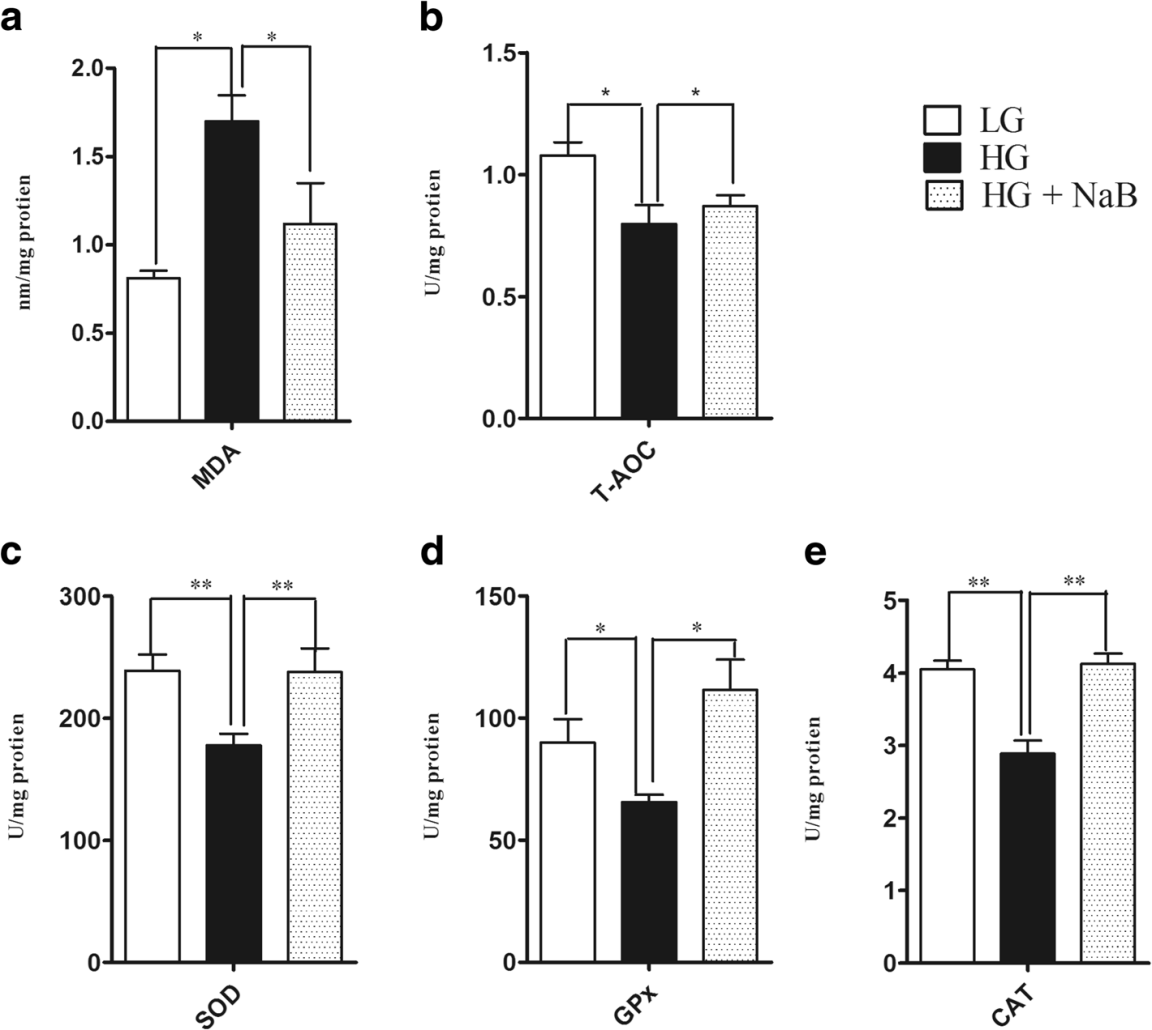
Effect sodium butyrate on lipid peroxidation, total antioxidant capacity and antioxidant enzyme activity in the liver of goats fed HG vs. LG and HG alone. **a** MDA. **b** T- AOC. **c** Total SOD activity. **d** GPx activity. **e** Catalase activity. The results are expressed as the mean ± SEM. Data were analysed by mixed model in SAS (SAS version 9.2, SAS Institute Inc., USA) and are expressed as the mean ± S.E.M. **P < 0.01, * *P* < 0.05 vs. LG. HG = high grain; LG = low grain; HG + NaB = high grain + sodium butyrate; MDA = malondialdehyde; T-AOC = total antioxidant capacity; TSOD = total superoxide dismutase; GPx = glutathione peroxidase; CAT = catalase

**Fig. 6:**
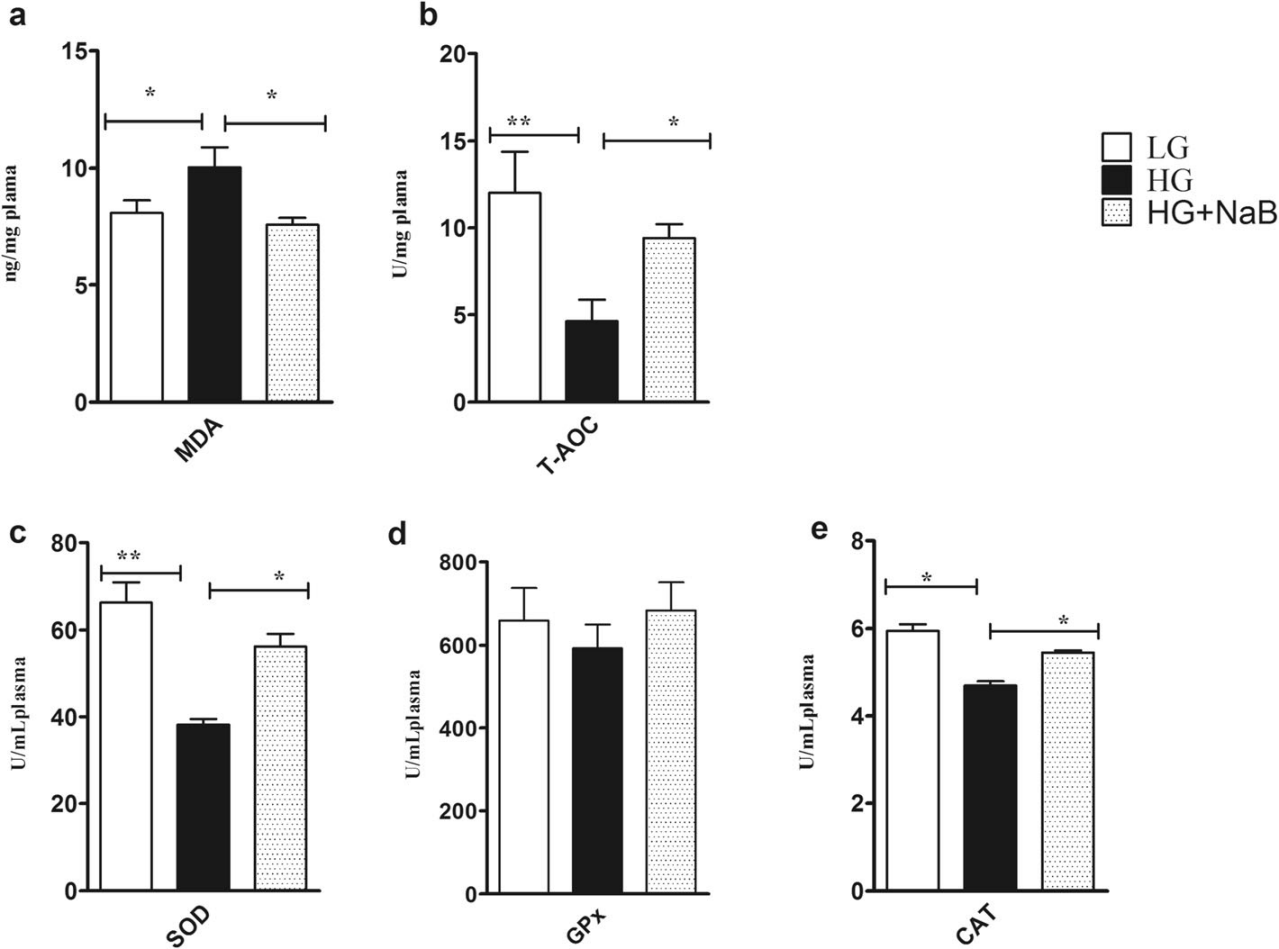
Effect of sodium butyrate on lipid peroxidation, total antioxidant capacity and antioxidant enzyme activity in the plasma of goats fed the HG compared with HG alone and LG . **a** MDA. **b** T-AOC. **c** Total SOD activity. **d** GPx activity. **e** Catalase activity. All enzyme activities and biomarkers were evaluated spectrophotometrically, and the results are expressed as the mean ± SEM. Data were analysed by mixed model in SAS (SAS version 9.2, SAS Institute Inc., USA) and are expressed as the mean ± S.E.M. **P < 0.01, * *P* < 0.05. HG = high grain; LG = low grain; MDA = malondialdehyde; T-AOC = total antioxidant capacity; TSOD = total superoxide dismutase; GPx = glutathione peroxidase; CAT = catalase

The values of T-AOC and MDA in both liver and plasma are shown in Figs. 5a, b and 6a, b. Feeding the HG + NaB diet significantly decreased MDA in the liver and plasma of lactating goats (*p* = 0.01 and *p* = 0.04, respectively) compared to feeding the HG diet. However, T-AOC was not reduced in a HG + NaB group (*p* = 0.03 and 0.02) compared to the HG group, whereas no differences were observed between the goats fed the LG and HG + NaB diets.

### Antioxidant enzyme activity

Variables related to antioxidant enzyme activity in the liver, including the concentrations of TSOD, GPx and CAT, showed significant differences (*p* = 0.005, *p* = 0.013, *p* = 0.001, respectively) among the goats fed the HG + NaB, HG and LG diets; however, no differences were observed between goats fed the LG and HG + NaB diets (Fig. 5c, d, e). Additionally, the results showed significant increases in TSOD and CAT activity in the plasma of goats fed the HG diet with NaB compared with the HG diet alone (*p* = 0.001 and *p* = 0.05, respectively). These findings were consistent with the liver antioxidant enzyme activity; however, no significant differences were observed in the plasma GPx activity among the groups (Fig. 6c, d, and e).

### Nrf2 protein expression

**Fig. 7:**
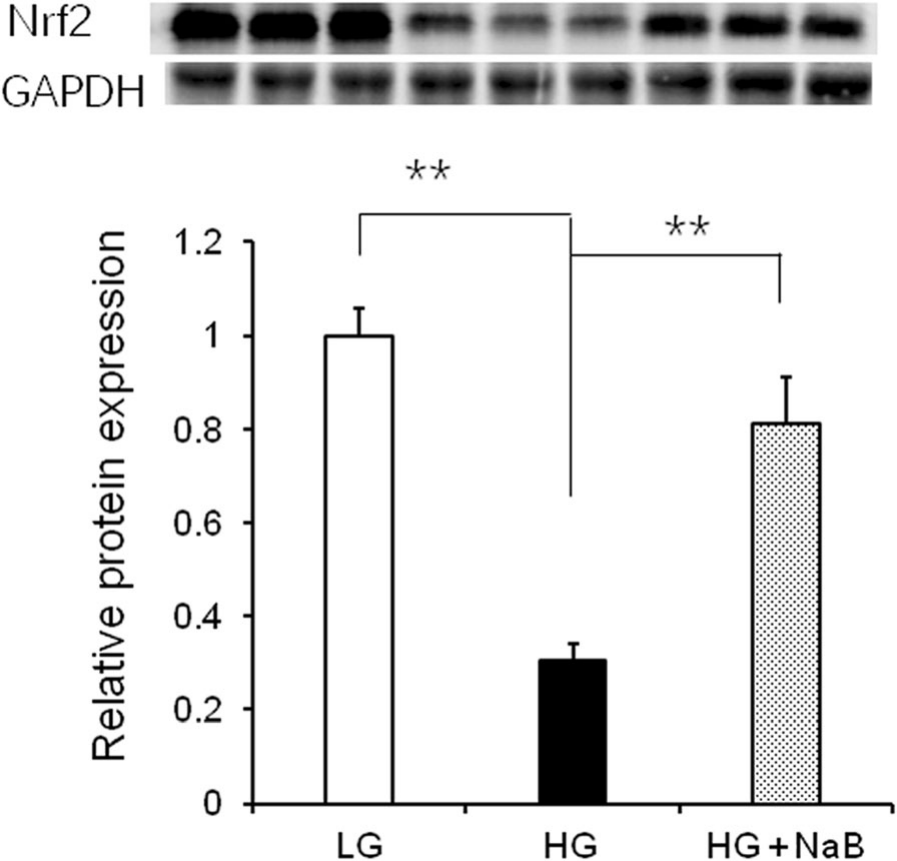
Effect of sodium butyrate on the relative expression of Nrf2 protein in the livers of goats fed the HG compared with the HG alone or LG. GAPDH was used as the reference protein. Nrf2 protein was significantly higher than in goats fed the high-grain diet. Data were analysed by mixed model in SAS (SAS version 9.2, SAS Institute Inc., USA) and are expressed as the mean ± S.E.M. **P < 0.01, * *P* < 0.05

As shown in Fig. 7, we observed significant differences in Nrf2 protein expression among the three groups (*p* = 0.01). Although the HG + NaB diet increased the levels of Nrf2 protein, the Nrf2 level still remained lower than in goats fed the LG diet.

## Discussion

In the present study, we determined the ability of sodium butyrate to mitigate the oxidative stress induced by long-term feeding of a high-grain diet in lactating dairy goats. In this study, we used three different feed formulas, low grain as a control, high grain to induce SARA, and high grain with sodium butyrate, to investigate the effect of NaB on SARA, and our results confirmed the presence of SARA in the group of goats fed a high-grain diet [33], in which ruminal pH fell below 5.8 for more than 180 min/d [34]. Moreover, our results showed lower levels of milk yield (kg/d), milk fat (%), and milk fat yield (kg/d) in goats fed HG alone, these have been previously reported by Dong et al., (2011) [35]. These milk composition reduced because the free circulating LPS may decrease the activity of key enzymes in milk component syntheses, such as acetyl-CoA carboxylase, fatty acid synthase, and lipoprotein lipase [15]. Consistent with previous SARA studies we observed increased levels of proinflammatory cytokines in goats fed HG alone [36] and conversely these proinflammatory cytokines were prominently decreased in goat fed HG with NaB. These further confirmed the presence of SARA in a group of goats fed high grain diets. Meanwhile, sodium butyrate successfully improves the rumen pH when added to the HG diets [37, 38]; this is contradictory to the results of ruminal volatile fatty acids which is significantly higher in goats fed HG with sodium butyrate. However it’s previously reported by Norton et al., that sodium butyrate infusion significantly increased rumen fluid VFA and pH [39], it is possible that butyrate altered the metabolic flux of the rumen, favoring the neutralization of H+ through the efflux of bicarbonate [40]. There are several conflicting results have been observed in previous studies about the effect of NaB on rumen pH; e, g., Laarman et al... demonstrated the presence of SARA in both groups of cows fed a total mixed ration and concentrate with or without NaB [41], whereas Herrick et al. reported a significant increase in rumen pH in lactating dairy cows ruminally dosed with butyrate [42]. Moreover, Schlau et al. observed a marked increase in the molar proportion of butyrate in acidosis-resistant steers compared to that in acidosis-susceptible steers and stated that this increase may have at least partially contributed to the higher pH observed in acidosis-resistant steers [37]. This discrepancy may be because of using different concentrations of infused butyrate. Consistent with these findings, lower levels of LPS in rumen fluid, portal vein plasma and hepatic vein plasma were observed in the current study. The present results are in agreement with those previously reported by Li et al [19]. Those results showed that exogenous butyrate had a stimulating effect on the native butyrate-producing bacterial population. This increase in butyrate-producing bacteria could maintain the stability of the fibre-degrading microbiota during SARA. Thus, the release of LPS in the rumen was reduced and gastrointestinal homeostasis was maintained. Furthermore, other studies demonstrated that butyrate maintains barrier function through the genomic regulation of several cellular processes, and it modulates gene expression through histone deacetylase complex (HDAC) inhibition [20], which in turn can reduce LPS production and prevent LPS translocation into the bloodstream, potentially reducing the systemic effect of LPS. Butyrate has been implicated in the down-regulation of bacterial virulence, both by direct effects on virulence gene expression and by acting on the proliferation of host cells [22]. Conversely consuming a HC diet leads to remarkable increases in LPS produced in the digestive tract and delivered into the liver via the portal vein [43]. The increased levels of LPS trigger a liver inflammatory response through the TLR4-signaling pathway, lead to an increase of pro- and anti-inflammatory cytokines and acute phase proteins (APPs) including LPS-binding protein (LBP), haptoglobin (Hp) and serum amyloid A (SAA) in the peripheral blood [13, 44]. This study demonstrated that supplementation of a high-grain diet (HG) with NaB promotes antioxidant activity and alleviates oxidative stress during SARA. The higher concentration of plasma and liver SOD, CAT and GPx in the goats fed the HG + NaB diet than in the goats fed the HG diet could be related to the higher ruminal pH and reduced levels of LPS in associated with the HG + NaB diet, and these factors may reduce the inflammatory reaction and reactive oxygen species (ROS) production. Moreover, among the possible other mechanisms by which butyrate may boost enzyme activity, antioxidant activity has also been recently suggested. Ma et al. reported significantly increased levels of antioxidant enzymes in piglet intestinal cells following butyrate incubation, and they concluded that the sodium butyrate-mediated alteration in antioxidant indices, including MDA, GSH, and antioxidative enzymes, suggests an improvement in the level of oxidative stress in the intestinal mucosa cells, which may result in improved wound healing, tight junctions, epithelial integrity and defence systems [45]. Furthermore, Dionissopoulos et al. reported high expression of GPX2 in cow rumen papillae after butyrate supplementation; GPX2 is known to protect the rumen from ROS produced by ruminal bacteria [40]. Moreover, several clinical studies have demonstrated that butyric acid or its sodium salt mediate immune responses and antioxidant capacity in vitro and in vivo [46, 47]. Butyrate can decrease oxidative stress and cytokine synthesis, even at very low doses [22]. Conversely, in goats fed the HG diet, we observed lower activity of antioxidant enzymes, including SOD and CAT, in both liver and plasma and lower activity of GPx in the liver, which corresponded to lower levels of mRNA. These results are in accordance with evidence previously reported by Watson et al., who studied the activities of conjugating and antioxidant enzymes following endotoxin exposure in male Sprague Dawley rats and concluded that total SOD activity and CAT and GPx activity were decreased at different time points following endotoxin administration [48].

In our results, the high-grain diet significantly increased liver and plasma MDA, which is in agreement with the results of Guo et al., who observed higher concentrations of plasma MDA in cows fed a diet containing 20% finely ground wheat (W20) than in cows fed 0% finely ground wheat (W0) [49]. On the other hand, the liver and serum MDA were significantly lower in the sodium butyrate group than in the high-grain diet group; this result was noted previously by Zhang et al. in stressed broiler chickens supplemented with microencapsulated NaB, and they concluded that NaB had partially elevated the oxidative stress and reduced the MDA levels [50]. A reduced level of MDA was also seen in intestinal cells treated with sodium butyrate accompanied by increased antioxidant enzyme indices [45]. Furthermore, Guo et al. reported decreased levels of MDA and increased levels of T-AOC and beta hydroxybutyric acid in cows with sub-acute ruminal acidosis regulated by pelleted beet pulp, which is a known butyrigenic carbohydrate source that may lead to increased levels of beta hydroxybutyric acid and alleviation of oxidative stress [49]. The increased levels of MDA and the decreased levels of T-AOC indicate that the pro-oxidants overwhelmed the antioxidants and exerted lipid peroxidation [51]. MDA is a highly toxic by-product generated by lipid peroxidation [52], and its toxicity depends on its rapid reaction with proteins and DNA [52]. If accumulated, these lipid peroxy radicals act on nearby fatty acids in the plasma membranes of the cells and induce radical formation by positive the feedback loop.

Nuclear factor E2-related factor-2 (Nrf2) is a transcription factor that orchestrates the expression of a battery of antioxidant and detoxification genes under both basal and stress conditions [53]. In our results, we observed up-regulation of UGT1A1, NQO1 and MGST3. These genes are known to be involved in butyrate-mediated defence against oxidative stress, providing evidence of favourable modulation of stress [54]. Butyrate was reported to have immune suppression [55] and anti-inflammatory properties, in part by suppressing NF-κB activity [56]. NF-κB is known to be antagonized by Nrf2. Recent studies indicated that sodium butyrate could attenuate inflammatory reactions by inhibiting the expression of inflammatory mediators such as NF-κB, tumour necrosis factor alpha (TNF-α) and interferon-gamma (IFN-γ) through modulation of antioxidant defence mechanisms [57]. These findings suggest that sodium butyrate could control the reactive oxygen species (ROS)-mediated activation of NF-κB. Furthermore, Yaku et al., (2012) [58] noted that sodium butyrate also causes enhancement of Nrf2 mRNA levels and suppression of p53 mRNA levels in normal intestinal epithelial cells, enhancing the activities of phase 2 enzymes via an increase in Nrf2 protein levels in the nucleus and a decrease in the mRNA and protein levels of p53.

## Conclusions

In conclusion, our study indicates that feeding a high-grain diet to lactating dairy goats reduces the mean ruminal pH. When added to the HG diet, sodium butyrate (NaB) increased the mean ruminal pH and reduced the levels of ruminal, portal and hepatic LPS, indicating that sodium butyrate reduced rumen fermentation and improved rumen tight junctions. Data from this study also demonstrate that sodium butyrate improved antioxidant capabilities to some extent due to increased mRNA expression of antioxidant genes, including the nuclear factor E2 related factor2 (*Nrf2*)-dependent genes SOD1, SOD2, SOD3, GPX1, CAT, UGT1A1, NQO1 and MGST3. Additionally, in goats fed the HG + NaB diet, TSOD and CAT enzyme activity and total antioxidant capacity (T-AOC) increased, and malondialdehyde (MDA) levels in both the liver and plasma decreased compared to goats fed the high-grain diet alone. However, GPx activity was increased only in the livers of goats in the HG + NaB group. Western blotting revealed increased total Nrf2 protein associated with the HG + NaB diet compared to the HG diet; however, Nrf2 levels remained lower than those in the LG diet. Taking all the above results together, this study indicates that sodium butyrate could improve the oxidative status in sub-acute ruminal acidosis through partial activation of a set of Nrf2-dependent genes.

## Abbreviations

CAT: Catalase
GAPDH: Glyceraldehyde 3-phosphate dehydrogenase
GPX: Glutathione peroxidase1
HG: High grain
HMOX2: Haem oxygenase 2
LG: Low gain
MGST3: Microsomal glutathione S-transferase 3
MT1A: Metallothionein 1A
MT2A: Metallothionein 2A
NaB: Sodium butyrate
NQO1: NAD(P)H, quinone 1;
Nrf 2: Nuclear factor E2-related factor 2
SOD: Superoxide dismutase
SRXN1: Sulfiredoxin 1
TXNRD1: Thioredoxin reductase 1
UGT1A1: UDP glucuronosyltransferase 1 family, polypeptide A1
